# Involvement of CD146 in the *Cryptococcus neoformans* adhesion and infection of brain endothelial cells

**DOI:** 10.1128/iai.00145-25

**Published:** 2025-04-10

**Authors:** Wei Liu, Junhong Chen, Ting Wang, Ying Gong, Chen Yang, Yuwei Li, Xiyun Yan, Hongxia Duan, Xiaoming Wang, Mingshun Zhang

**Affiliations:** 1 Department of Immunology, National Vaccine Innovation Platform, NHC Key Laboratory of Antibody Technique, Jiangsu Key Laboratory of Pathogen Biology, School of Basic Medical Sciences, Nanjing Medical University 271667, Nanjing, Jiangsu, China; 2 Nanozyme Laboratory in Zhongyuan, Henan Academy of Innovations in Medical Science, Zhengzhou, Henan, China; 3 CAS Engineering Laboratory for Nanozyme, Key Laboratory of Biomacromolecules, Institute of Biophysics, Chinese Academy of Sciences, Beijing, China; NIH, NIAID, Washington, DC, USA

**Keywords:** *Cryptococcus neoformans*, blood-brain barrier, endothelial cells, CD146, neutralization

## Abstract

Cryptococcal meningitis is a common and refractory central nervous system (CNS) infection with high mortality and disability. For *Cryptococcus neoformans* (*C. neoformans*) to penetrate the CNS, it first adheres to and breaches the blood‒brain barrier (BBB). Here, we explored the roles of CD146, an adhesion molecule expressed on the surface of brain microvascular endothelial cells (BMECs), in cryptococcal vascular adhesion and BBB invasion. Following cryptococcal infection, we observed a reduction in CD146 expression in BMECs, which was at least partially mediated by metalloproteinase-9. Once overexpressed on BMECs, CD146 increased *C. neoformans* adhesion; in contrast, CD146 knockout decreased the attachment of fungi to endothelial cells *in vitro*. Unexpectedly, CD146 knockout failed to reduce fungal infection in the brain following intravascular instillation of *C. neoformans*. However, *the* anti-CD146 antibody AA98 significantly increased the fungemia (spleen CFU), suggesting that CD146 may be involved in the early adhesion and invasion of Cryptococcus into cerebral vessels. AA98, however, failed to extend the survival of *C. neoformans* infected mice. These results suggest that CD146 may play dispensable roles in the *C. neoformans* brain infection.

## INTRODUCTION

Cryptococcal meningitis is a central nervous system (CNS) infection caused by invasion of the meninges and/or brain parenchyma by *Cryptococcus neoformans* (*C. neoformans*) (1, 2). It is characterized by severe intracranial hypertension and damage to the brain parenchyma, and its onset is insidious (3). This opportunistic infection is common in people with human immunodeficiency virus (HIV) infection, organ transplant recipients, or other immunocompromised conditions, as well as in a small proportion of immunocompetent persons (4–6). *C. neoformans* is an opportunistic pathogenic yeast that widely exists in the natural environment (7, 8). After being inhaled through the respiratory tract, *C. neoformans* can proliferate within the lungs and disseminate to the brain via the blood circulation, and meningitis can be lethal if *C. neoformans* penetrates through the blood‒brain barrier (BBB) and colonizes the CNS (9, 10). The BBB is created by the endothelial cells that form the walls of the capillaries, and the tight junctions between the endothelial cells form an effective seal (11). Therefore, the adhesion of *C. neoformans* cells to brain microvascular endothelial cells (BMECs) is one of the first steps of para- or trans-cellular brain invasion (12).

Cluster of differentiation 146 (CD146) was originally identified as a melanoma cell adhesion molecule and is highly expressed at the intercellular junctions of endothelial cells (13, 14). CD146 was demonstrated to be involved in bacterial adherence to the asthmatic airway epithelium (15). Recently, we demonstrated that CD146, an adhesion molecule, mediated the adhesion of *C. neoformans* to the respiratory epithelium (16). Moreover, CD146 in macrophages regulates pulmonary Cryptococcus infection (17). However, the roles of CD146 in *Cryptococcal* meningitis are still elusive.

In the present study, we aimed to investigate the roles of CD146 in the adhesion of *C. neoformans* to cerebral vessel endothelial cells and *Cryptococcal* meningitis. Our data demonstrated that *C. neoformans* decreased CD146 on brain endothelial cells via metalloproteinase-9 (MMP-9). Upregulating CD146 expression increased *C. neoformans* adherence to BMECs, whereas CD146 knockout decreased this adhesion *in vitro*. However, CD146 deficiency was dispensable for *C. neoformans* brain infection *in vivo*. Although treatment with the anti-CD146 antibody AA98 decreased the fungemia, AA98 failed to extend the survival of *C. neoformans* infected mice. These findings suggest that CD146 may function as a mediator of BBB invasion by free *C. neoformans*. Nevertheless, CD146 in the progression of cryptococcal meningitis is dispensable.

## RESULTS

### *C. neoformans* reduces CD146 expression in brain endothelial cells

**Fig 1:**
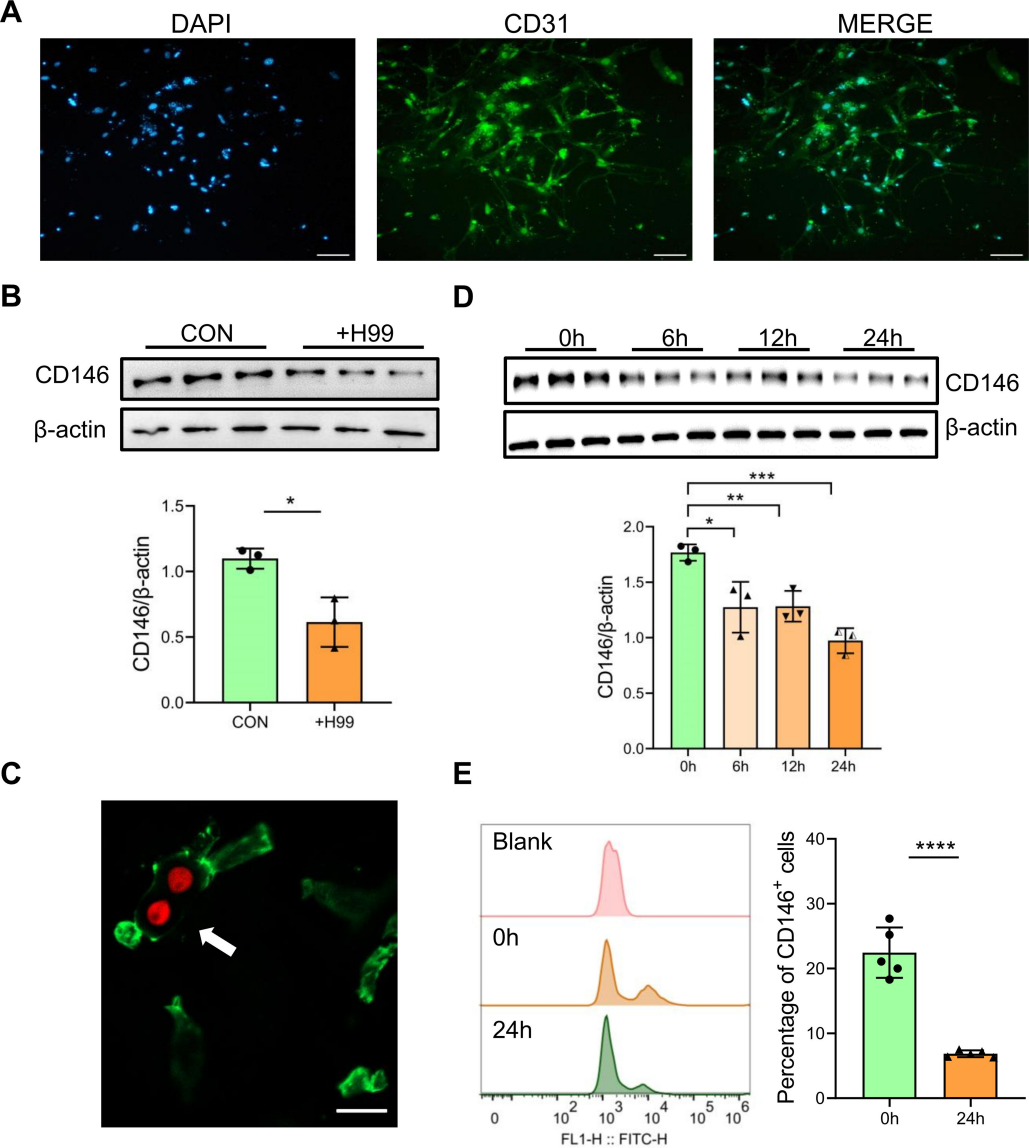
*C. neoformans* infection decreased CD146 on brain endothelia l cells. (**A**) Primary microvascular cerebral endothelial cells from C57BL/6J mice were extracted, grown into a single layer in 24-well plates, and identified with anti-CD31, an endothelial cell surface marker. (**B**) Western blotting analysis of CD146 on the primary microvascular cerebral endothelial cells from the mice with or without the *C. neoformans* infection. (**C**) C57BL/6J mice were injected with *C. neoformans* (20 × 10^6^ tdTomato-expressing strain H99) via the tail vein. Twenty-four hours later, the mice were anesthetized, and perfusion was performed. The brain was removed from frozen sections as described in the Materials and Methods. The microvascular cerebral vasculature was labeled with anti-CD146 (green). A white arrow points to a decline in CD146 protein fluorescence staining around blood vessels attached by Cryptococcus. Scale bar: 10 µm. (**D**) Western blotting was used to examine CD146 in the murine brain endothelial cell line bEnd.3 after 0, 6, 12, and 24 hours of infection with strain H99. (**E**) Flow cytometry analysis of CD146 expression on bEnd.3 cells treated with H99 before and 24 hours after infection. Each column represents the mean ± SEM of multiple replicates. *P < 0.05; **P < 0.01; ***P < 0.001; ****P < 0.0001; and ns, not significant.

First, we isolated and cultured brain microvascular primary endothelial cells. The surface marker CD31 in vascular endothelial cells was identified via immunofluorescence staining (Fig. 1A). Following 24 hours of *C. neoformans* strain H99 infection, primary brain endothelial cells significantly decreased CD146 expression (Fig. 1B). Similarly, the CD146 protein level in infected areas was reduced visually in the mouse cerebrovascular system following 24 hours *of i.v.* infection with H99 cells (Fig. 1C; Fig. S1). To further support that *C. neoformans* reduced CD146 in brain endothelial cells, we infected the murine brain endothelial cell line bEnd.3 with H99 for 0, 6, 12, or 24 hours. As expected, CD146 expression gradually decreased following *C. neoformans* infection (Fig. 1D). Additionally, after 24 hours of H99 cell stimulation, the mean fluorescent intensity of CD146 and the number of CD146^+^ cells were decreased, as confirmed by flow cytometry (Fig. 1E). Overall, CD146 was significantly decreased in brain endothelial cells infected with *C. neoformans*, suggesting that CD146 may be involved in cryptococcal vascular adhesion and infection.

### *C. neoformans* cleaves CD146 from brain endothelial cells via MMP-9

**Fig 2:**
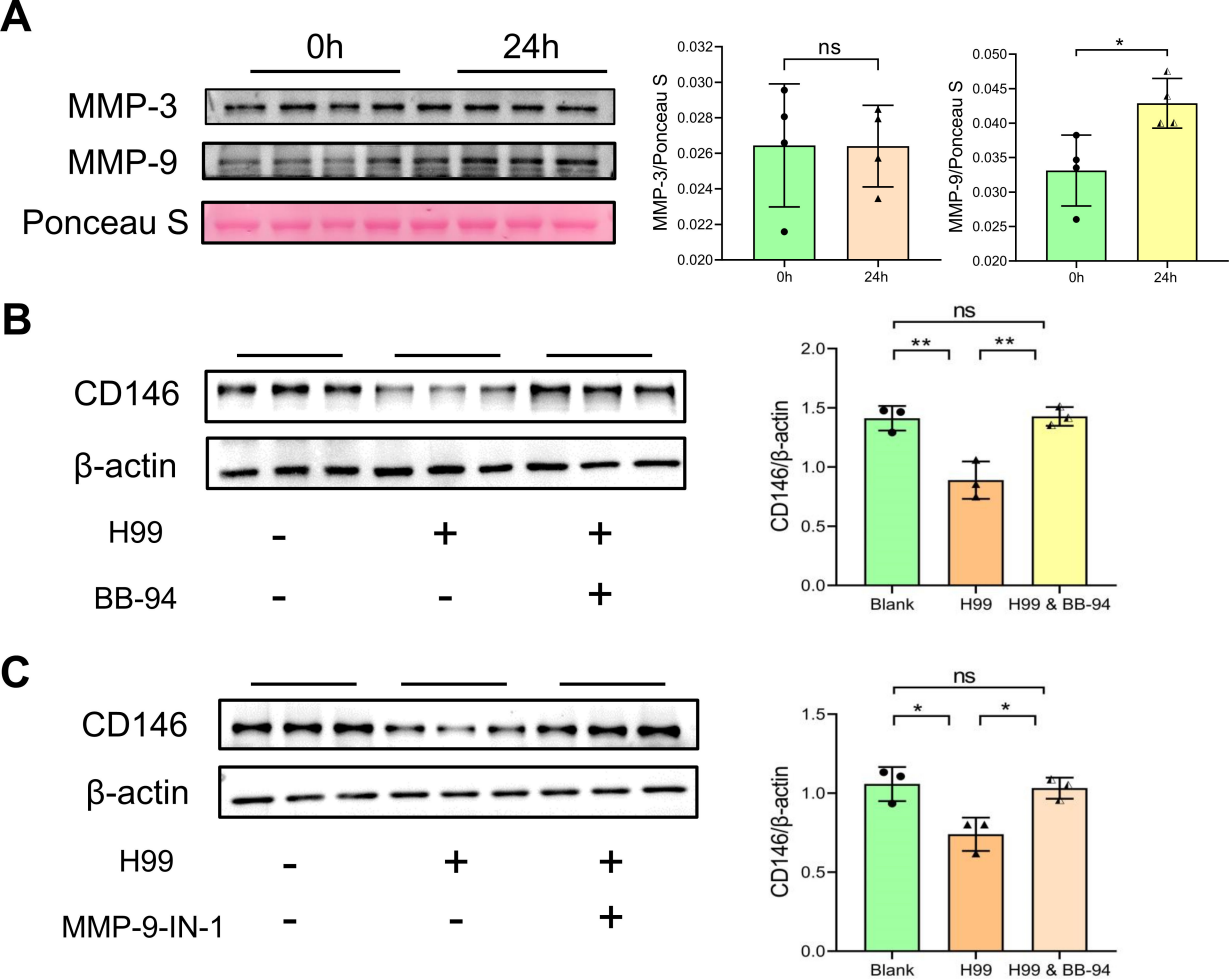
Inhibiting the metalloprotease secreted by *C. neoformans* rescued the expression of CD146 in the brain endothelium. (**A**) After bEnd.3 cells were stimulated with 2 × 10^6^ H99 cells per well for 24 h, secreted MMP-3/9 in the cell culture supernatant was detected by Western blotting, and Ponceau S staining was used as a loading control to assess the level of total protein. (**B, C**) A broad-spectrum MMP inhibitor, BB-94, or a specific MMP-9 protease inhibitor, MMP-9-IN-1, was simultaneously added to the fungal infection cell model shown in Figure A, and CD146 expression in endothelial cells was measured by Western blotting. Each column represents the mean ± SEM of multiple replicates. *P < 0.05; **P < 0.01; ns, not significant.

The shedding of CD146 on microvascular endothelial cells is linked to the synthesis of matrix metalloproteinases (MMPs) (18, 19), and the secretion of metalloproteases also contributes to the ability of *C. neoformans* to invade the CNS (20). Therefore, we hypothesized that *C. neoformans* cleaves the CD146 protein from endothelial cells through a metalloprotease. In particular, MMP-3 may be involved in CD146 shedding (19), and studies in patients with cryptococcosis showed high levels of MMP-9 in the cerebrospinal fluid of patients infected with *C. neoformans* (21). Therefore, we focused on the MMP-3 and MMP-9. Following 24 hours of H99 cell stimulation of bEnd.3 cells, the protein levels of MMP-3 and MMP-9 in the supernatant were measured by Western blotting. We discovered that MMP-9 secretion increased in the supernatant, while there was no discernible change in MMP-3 expression (Fig. 2A). We verified that the broad-spectrum MMP protease inhibitor BB-94 and the specific MMP-9 protease inhibitor MMP-9-IN-1 both rescued the Cryptococcus-induced decrease in CD146 protein expression in brain endothelial cells (Fig. 2B through E). These findings suggested that *C. neoformans* cleaved CD146 from the vascular endothelium, which at least partially was mediated by MMP-9 from infected endothelial cells.

### CD146 promoted *C. neoformans* adhesion to brain endothelial cells

**Fig 3:**
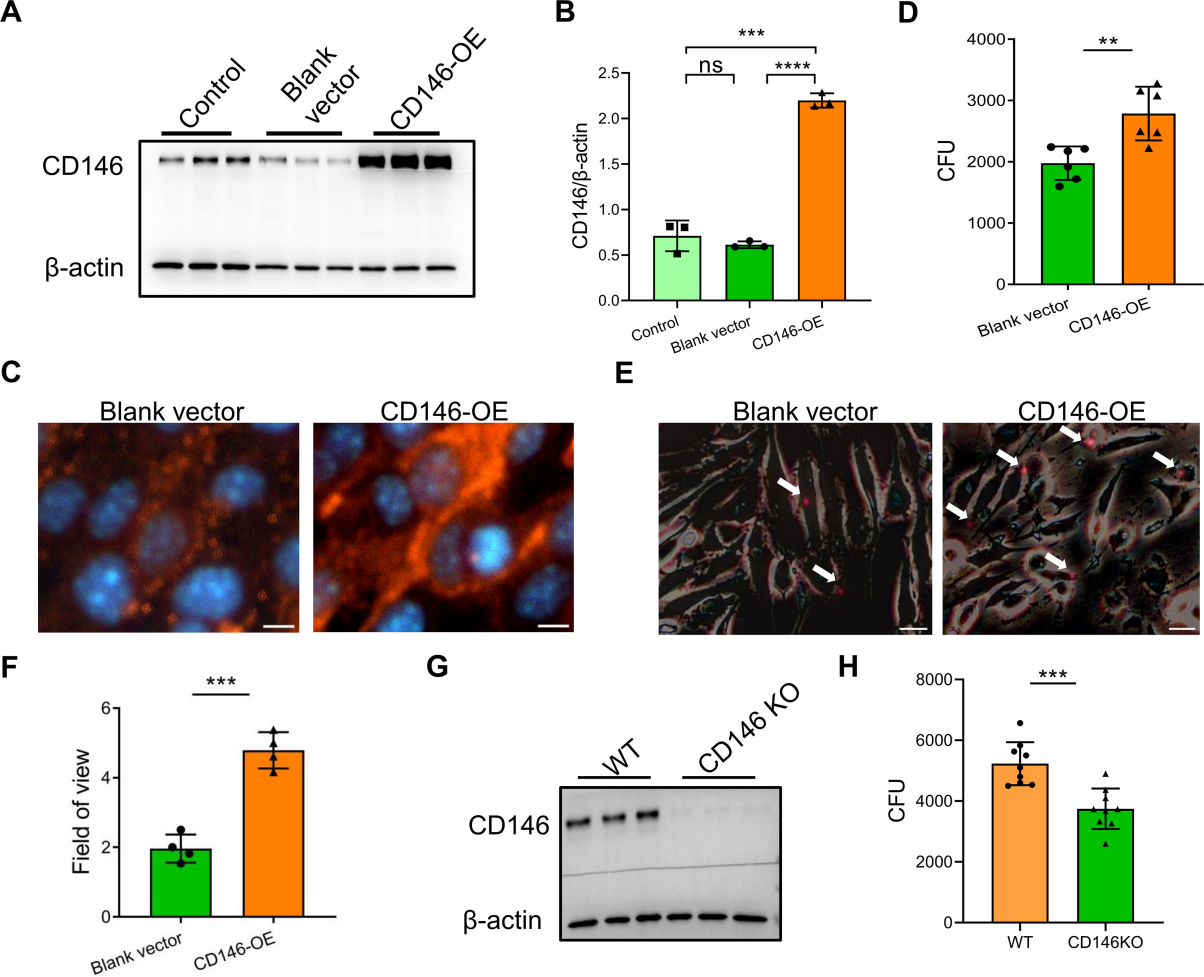
CD146 facilitates the binding of *C. neoformans* H99 to murine brain endothelial cells. (**A–C**) CD146 overexpression in bEnd.3 cells was verified by Western blotting and immunofluorescence analysis. For Fig. C, CD146 protein in tangerine, nuclei in blue. (**D**) bEnd.3 cells overexpressing CD146 were exposed to H99 for 4 hours at 37°C. The rate of association was determined by live CFU counts. (**E**) The increase in the number of bound tdTomato-expressing cryptococci was determined via fluorescence microscopy. (**F**) The average number of tdTomato-expressing H99 adherents per 10 endothelial cells in the field of visual field. Scale bar = 20 µm. (**G**) CD146 deficiency in the brain primary endothelial cells of the CD146-KO mice was detected by Western blotting. (**H**) CD146-knockout bEnd.3 cells were exposed to H99 for 4 hours at 37°C. The rate of association was determined by live CFU counts. Each column represents the mean ± SEM of multiple replicates. *P < 0.05; **P < 0.01; ***P < 0.001; ****, *P* < 0.0001; and ns, not significant.

Thus far, we reported that *C. neoformans* decreased CD146 in brain endothelial cells via MMP-9. To explore whether CD146 is involved in *C. neoformans* adhesion to brain endothelial cells, we overexpressed CD146 in bEnd.3 cells. Western blotting and immunofluorescence were used to confirm the overexpression of CD146 (Fig. 3A and B). In addition to CD146 elevation, the number of affixed cryptococci was significantly increased, and the viability of adhering cryptococci was determined via colony-forming unit (CFU) assays (Fig. 3C and D). Similarly, this increased number of strongly attached tdTomato-expressing cryptococci was directly observed via fluorescence microscopy (Fig. 3E and F). In contrast, CD146 deficiency in brain endothelial cells decreased the adhesion of *C. neoformans* (Fig. 3G and H). Collectively, these data indicate that CD146 may facilitate the adhesion of cryptococci to cerebral vessels.

### CD146 deficiency was dispensable for *C. neoformans* brain infection

**Fig 4:**
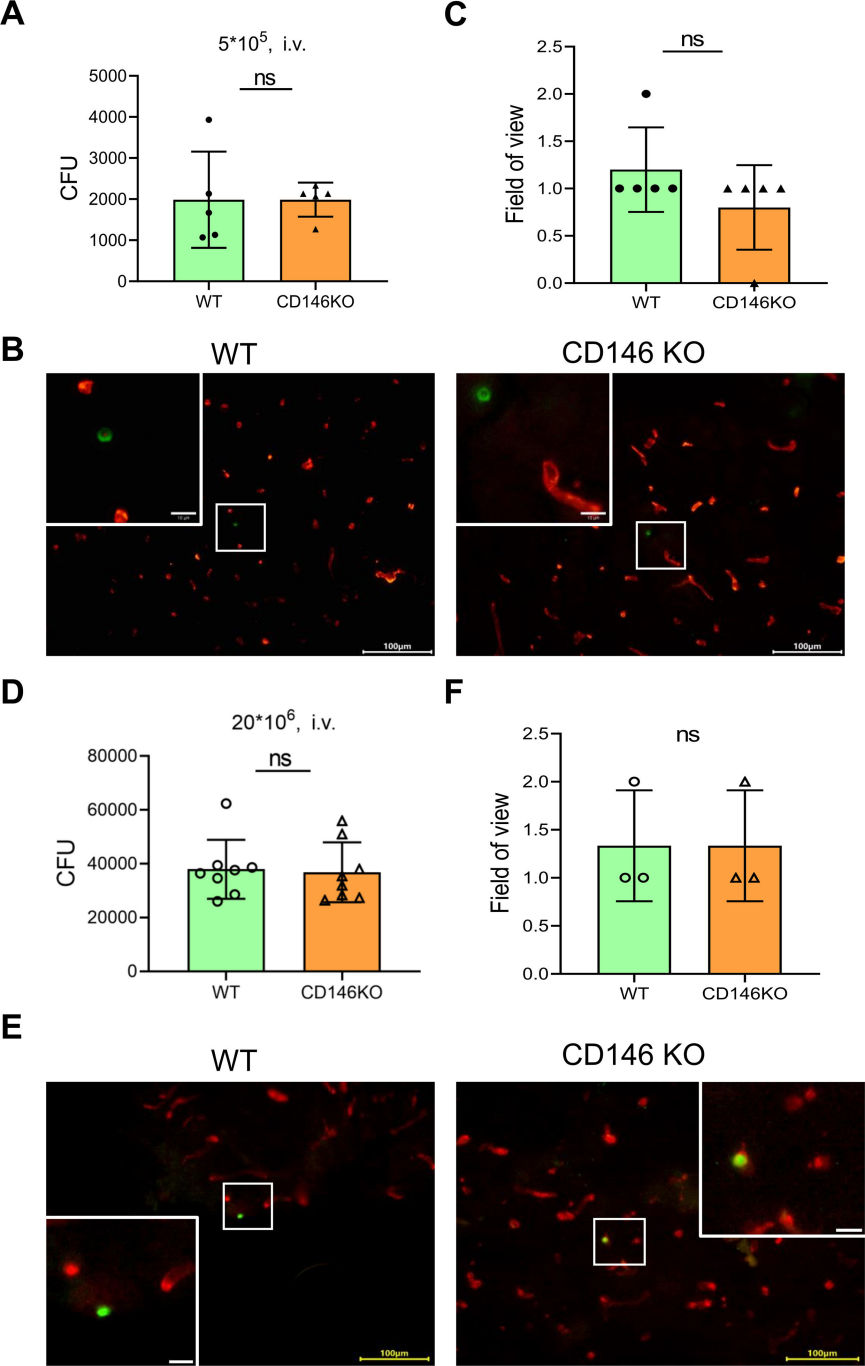
The adherence of *C. neoformans* to brain endothelial cells was not affected by the absence of CD146 *in vivo*. WT and CD146-KO mice were injected with 5 × 10^5^ (low-dose) *C. neoformans* H99 via the tail vein and sacrificed 24 hours after inoculation. (**A**) Brain CFUs were enumerated post infection by plating organ homogenates on Sabouraud dextrose agar. (**B**) Immunohistochemistry was performed post infection to label *C. neoformans* (green) and vessels (red). Squares represent areas enlarged in insets. (**C**) Field of view (FOV) showing yeasts located in the parenchyma of the brain. Scale bar = 100 µm. The mice were injected with 20 × 10^6^ (high-dose) *C. neoformans* H99 via the tail vein and sacrificed 24 hours after inoculation. (**D**) Brain CFUs were enumerated post infection by plating organ homogenates on Sabouraud dextrose agar. (**E**) Immunohistochemistry was performed post infection to label *C. neoformans* (green) and vessels (red). Squares represent areas enlarged in insets. (**F**) The FOV describes yeasts located in the parenchyma of the brain. Scale bar = 100 µm. Each column represents the mean ± SEM of multiple replicates. ns, not significant.

To investigate whether CD146 deficiency affects fungal invasion into the CNS, wild-type (WT) and CD146-knockout (CD146-KO) mice were *i.v*. injected with 5 × 10^5^ (low-dose) or 20 × 10^6^ (high-dose) *C. neoformans* strain H99. We assessed the number of CFUs in the brains of the mice after 24 hours of infection, and there was no significant difference in the CD146-KO mouse brain fungal burden compared with that of the WT mice (Fig. 4A and D). To provide additional evidence, immunofluorescence staining was used to detect the number of yeasts trapped in the parenchyma of the brain after injection, and there were also no noticeable changes (Fig. 4B, C, E and F). In summary, unlike *in vitro* findings that CD146 mediated fungal adhesion to brain endothelial cells, CD146 deficiency does not affect cryptococcal brain invasion or brain fungal burden.

### Blocking CD146 failed to treat *C. neoformans* infection

**Fig 5:**
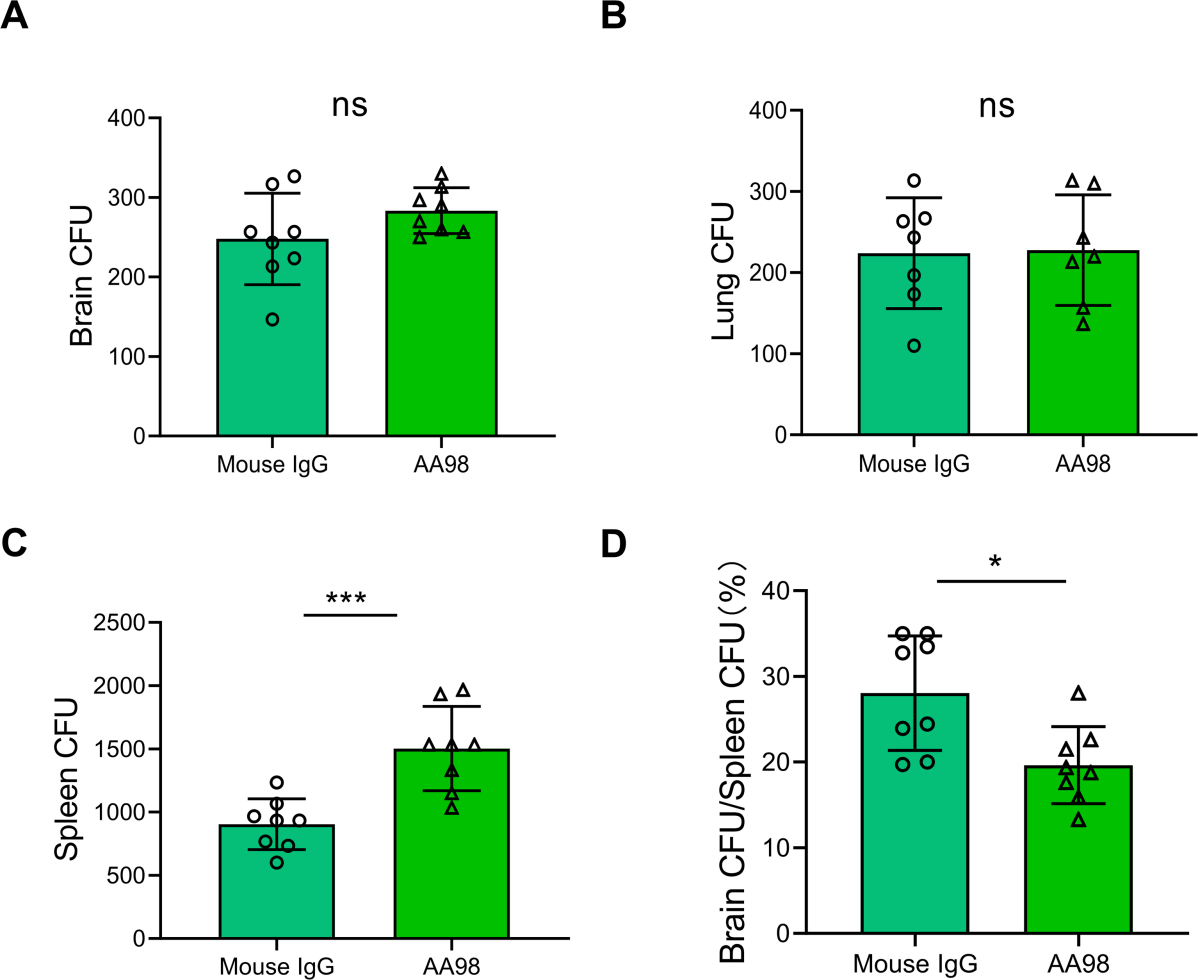
Treatment with anti-CD146 neutralizing antibodies *in vivo* decreased the transmission of *Cryptococcus* from peripheral immunological organs to the brain. WT mice were intraperitoneally injected with the CD146 neutralizing antibody AA98 or with isotype IgG 1 hour before being *i.v*. injected with 10 × 10^6^ *C. neoformans* strain H99. (**A–C**) Lung, spleen, and brain CFUs were enumerated 24 hours post infection by plating organ homogenates on Sabouraud dextrose agar. (**D**) Ratios of fungal loads measured in the brain and spleen. Each column represents the mean ± SEM of multiple replicates. *P < 0.05; **P < 0.01; ***P < 0.001; and ns, not significant.

**Fig 6:**
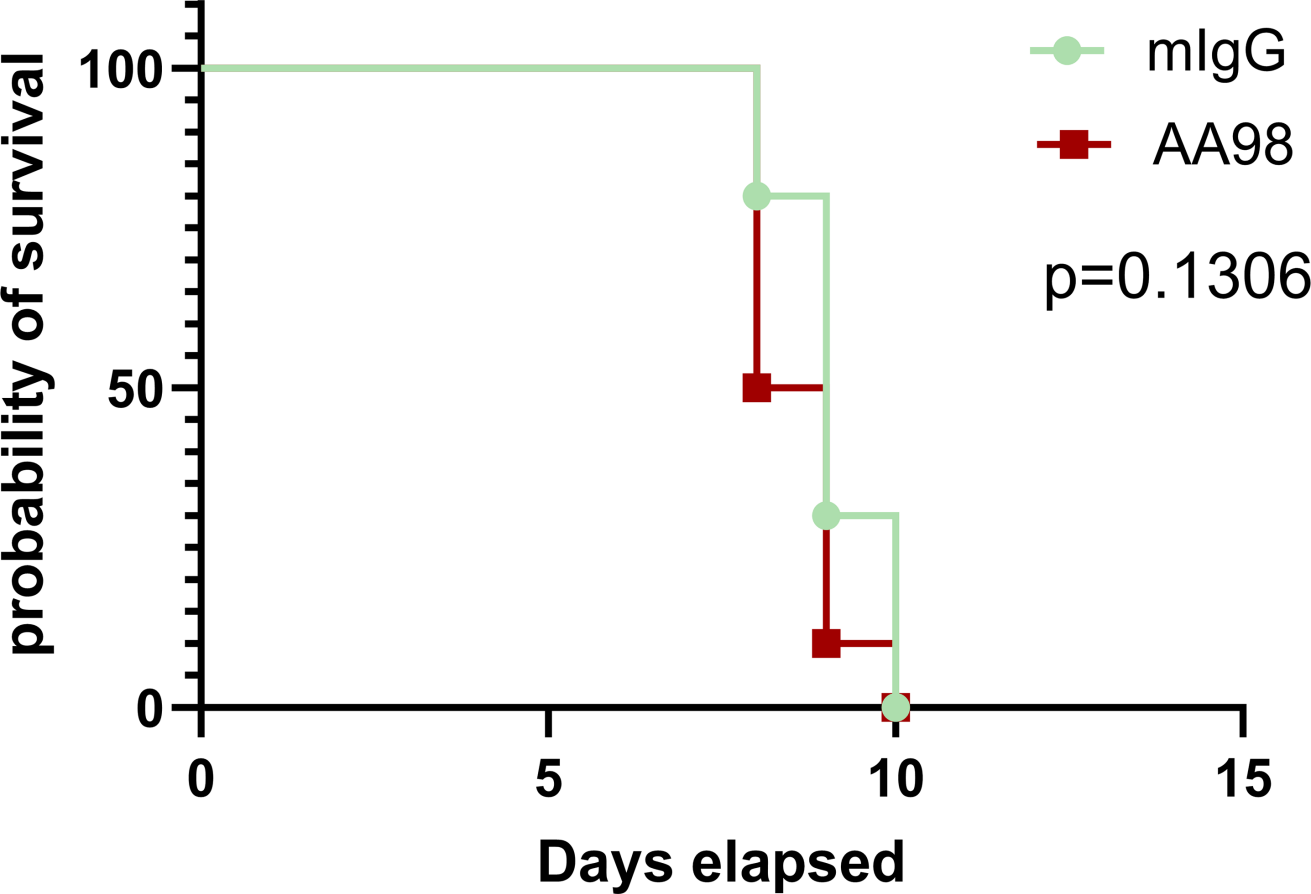
CD146 neutralizing antibody AA98 failed to extend the survival of *C. neoformans* infected mice. The mice were intraperitoneally injected with the CD146 neutralizing antibody AA98 or with isotype IgG. After 1 hour, these mice were intravascularly injected with 10 × 10^6^ *C. neoformans* strain H99. Ten mice were infected for each group. The survival study comparison was performed using the Log-rank (Mantel-Cox) test.

Thus far, CD146 deficiency does not seem to affect fungal invasion into the brain. As a molecule beyond adhesion molecules, CD146 can regulate the differentiation and development of vascular pericytes and maintain the integrity of the BBB (22). CD146 deficiency compromises the integrity of the BBB (23–25), which may promote fungal BBB transmigration and brain infection. We speculated that blocking CD146 may decrease the adherence of cryptococci to brain vessels. To test this hypothesis, we administered the CD146 neutralizing antibody AA98 to the mice via intraperitoneal injection 30 minutes before infection, whereas the control mice were administered the same volume of isotype IgG. The mice were then *i.v*. injected with 10 × 10^6^ *C. neoformans* strain H99, and after 24 hours of infection, the fungal burdens in the brain, lung, and spleen were determined. The results revealed that there was no significant difference in the number of CFUs in the brain or lung tissue between the control group and the CD146 neutralizing treatment group (Fig. 5A and B), whereas a progressive increase in the splenic fungal burden was observed in the CD146 neutralizing treatment group (Fig. 5C). Fungemia is a prerequisite for *C. neoformans* brain infection (26). The presence of *C. neoformans* in the spleen may indicate the degree of fungemia. We compared the ratios of the fungal burden in the brain and spleen, an indicator of the invasion of Cryptococcus into brain tissue. Compared with the control antibody, AA98 blocked CD146 and reduced the invasion of *C. neoformans* from the spleen to the brain (Fig. 5D). Furthermore, we explored whether AA98 could extend the survival time of the mice with *C. neoformans* fungemia. Unfortunately, AA98 failed to prolong the survival time of the *C. neoformans* infected mice (Fig. 6). Overall, CD146 may be dispensable for the *C. neoformans* brain infection.

## DISCUSSION

Cryptococcal meningitis, a lethal illness caused by the fungus *C. neoformans,* is the second leading cause of HIV-related mortality worldwide (27, 28). Previous evidence has shown that the initial site of cryptococcal entry into the brain is the endothelial BBB (2) and that CD146 is involved in the regulation of BBB development (22). Our present study revealed a decrease in CD146 expression in BMECs following cryptococcal infection, which may be due to the cleavage of metalloprotease. CD146 on BMECs mediated the adhesion of *C. neoformans*. Unexpectedly, CD146 knockout or blocking CD146 with the neutralizing antibody AA98 seemed to be dispensable for *C. neoformans* infection in the brain.

As an adhesion molecule on endothelial cells, CD146 may harness multiple ligands, i.e., netrin-1 to promote angiogenesis and vascular development (14). Moreover, CD146 is implicated in the virus, bacterial, and fungal infectious diseases (15–17, 29). Specifically, CD146 mediates the adhesion of Mycoplasma pneumoniae, nontypeable Haemophilus influenzae, and *C. neoformans* to airway epithelium (15, 16). As similar, the present study revealed that CD146 was essential for the adhesion of *C. neoformans* to brain endothelial cells *in vitro*. CD44, interacting with fungi hyaluronic acid, is the primary receptor for the adhesion of free *C. neoformans* to brain endothelial cells (30). In addition to hyaluronic acid, *C. neoformans* express Cpl1 (31) and other surface molecules (32). To effectively inhibit the adhesion of *C. neoformans* via CD146, we should clarify the fungi CD146 ligand(s).

Nevertheless, we did not find any decrease in cryptococcal vascular adherence in CD146 KO mice, as expected. Compared with those in WT mice, there was no discernible variation in the fungal burden in the brain in either the high-dose or low-dose fungemia infection model. The intricate outcomes *in vivo* are explained below. The deletion of CD146 on endothelial cells may reduce the adherence of *C. neoformans* to the vascular endothelium. However, owing to the increased permeability of the vascular endothelium after CD146 deletion (22), free cryptococci are more likely to migrate across the compromised BBB into the brain parenchyma. According to previous reports, endothelial CD146-deficient mice exhibit normal physiological angiogenesis development (23) but increased vascular permeability (24, 25). Collectively, CD146 knockout may decrease *C. neoformans* adhesion to brain endothelium but increase the fungi transmigration across compromised BBB, leading to a balanced fungi burden in the brain. Moreover, several *in vivo* studies *have* demonstrated that CD146 deficiency leads to decreased neutrophil recruitment after *M. pneumoniae* lung infection (33), whereas intravascular clearance of *C. neoformans* is dependent on neutrophil killing activity (34–36). Thus, we speculated that the decreased recruitment of neutrophils in CD146-KO mice resulted in a reduction in intravascular clearance of *C. neoformans*, which ultimately led to equivalent fungal burdens in the brain parenchyma compared with those in WT mice.

Furthermore, we used the neutralizing antibody AA98 to treat cryptococcal brain infection. Repeatedly, we observed that AA98 increased the spleen CFU, suggesting that AA98 may increase fungemia. However, the fungal burdens in the brain and lung tissues were unchanged with or without AA98 therapy. More importantly, AA98 failed to extend the survival time of *C. neoformans* infected mice. Therefore, CD146 may mediate the early attachment of free *C. neoformans* to the brain vascular endothelium. However, the roles of CD146 in *C. neoformans* pathogenesis may be dispensable.

Our study is not without limitations. Evidence from scanning electron microscopy (SEM) suggests that a metalloprotease secreted by *C. neoformans* could cause extensive microvillus-like structure alterations at the fungus‒endothelium interface (20). This may involve subtle changes in the CD146 protein, and *C. neoformans* may employ cleaved CD146 to masquerade as an internal molecule to better adhere to blood vessels. In the present study, we focused only on CD146 in endothelial cells. CD146 in macrophages (37, 38) and other immune cells, i.e., Th17 cells (39), warrants future research on *C. neoformans* brain dissemination. Either CD146 knockout or CD146 neutralization antibody AA98 not only targets brain endothelial cells but also influences other cells. Therefore, CD146 endothelial knockout or neutralization may work better to elucidate the roles of CD146 on endothelial cells and *C. neoformans* brain infection

In summary, we demonstrated that CD146 expression was decreased in mouse brain vascular endothelial cells following infection with *C. neoformans* both *in vivo* and *in vitro*. CD146 mediated the adhesion of *C. neoformans* to the brain endothelial cells *in vitro*. Unexpectedly, we failed to uncover the significance of CD146 *in vivo*. Neither CD146 knockout nor CD146 neutralization antibody AA98 decreased *C. neoformans* brain infection. Further studies aiming to specifically intervene in the interactions between CD146 and *C. neoformans* may help to overcome brain cryptococcosis.

## MATERIALS AND METHODS

### Animals

Female 6- to 8-week-old C57BL/6J mice were purchased from the Laboratory Animal Center, Nanjing Medical University (Nanjing, China). CD146^−/−^ (CD146-KO) mice generated via CRISPR/Cas9 techniques on the C57BL/6J background were obtained from Cyagen (Suzhou, China). The mice were maintained under specific pathogen-free (SPF) conditions at the Animal Core Facility of Nanjing Medical University. All animal treatments were in accordance with the guidelines approved by the Institutional Animal Care and Use Committee of Nanjing Medical University (IACUC-1708004).

### C. neoformans

The encapsulated strain H99, serotype A (catalog no. 208821), was obtained from the ATCC. The tdTomato-expressing H99 strain was generated and provided by Dr. Xiaorong Lin (University of Georgia). The fungi maintained in liquid nitrogen in 25% glycerol were grown to log phase in Sabouraud’s dextrose broth (Becton Dickinson, 238230) plates at 32°C with gentle rotation for 16 hours and washed three times in sterile PBS (pH 7.4) before use.

### Isolation and cultivation of murine brain endothelial cells

Six- to eight-week-old mice were sacrificed, and their brains were extracted. After the cerebella, striata, optic nerves, and white matter were removed, the cerebral cortices were collected. The tissue samples were digested in 15 mL of 0.1% collagen B (Roche, Indianapolis, IN, USA) supplemented with 30 U/mL DNase I (Sigma, St. Louis, MO, USA) for 1.5 h at 37°C and shaken every 30 minutes. The microvessel pellets were resuspended in medium containing 3 ng/mL 30% FBS, 10 U/mL heparin, 100 U/mL penicillin, 100 µg/mL streptomycin, and bovine fibroblast growth factor (Peprotech, Rocky Hill, NJ, USA). The microvessel suspensions were placed in 24-well plates precoated with rat-tail collagen I (Sigma-Aldrich) and incubated in 5% CO_2_ at 37°C. The medium was changed within the first 24 h and then every 2 days. After growing for 7–10 days, the endothelial cells reached confluence.

### Cell culture

The bEnd.3 cell line, an immortalized mouse brain endothelial cell line, was obtained from ATCC (CRL-2299). bEnd.3 cells were grown in DMEM (Gibco, USA) supplemented with 10% fetal bovine serum (FBS; Gibco, USA), 100 units/mL penicillin, and 100 µg/mL streptomycin (Yeasen Biotechnology, China). bEnd.3 cells were maintained in a humidified incubator at 37°C with 5% CO_2_ and 95% air. All experiments were carried out when the density of the cells was 90%–100%.

### Western blot analysis

Total protein was extracted in RIPA buffer (89900, Pierce) supplemented with PMSF (ST506, Beyotime, China) on ice and centrifuged for 10 minutes at 12,000 rpm at 4°C.

The supernatant was then transferred to a new tube and denatured in sodium dodecyl sulfate‒polyacrylamide gel electrophoresis (SDS‒PAGE) loading buffer (20315, Yeasen) with heating at 100°C for 10 minutes. The supernatant was then stored at –80°C. The proteins were separated by 10% SDS‒PAGE and transferred to polyvinylidene fluoride membranes (Millipore, Billerica, USA). The membranes were blocked for 1 h in 5% skim milk at room temperature (RT) and incubated at 4°C overnight with the following primary antibodies: anti-CD146 (81701S, Cell Signaling Technology, USA), anti-β-actin (GB15003, Servicebio), and anti-MMP-9 (ab228402, Abcam) overnight before the addition of HRP-linked goat anti-rabbit IgG (EarthOx Life Sciences, CA). After the membranes were treated with Immobilon Western Chemiluminescent HRP Substrate (WBKLS0500, Merck Millipore, USA), the binding of specific antibodies was visualized via a Syngene G:BOX Imaging System and analyzed with ImageJ. For proteins in the supernatant, Ponceau S (P0022, Beyotime, China) was used as the loading control.

### Immunofluorescence staining

Twenty-four hours after inoculation with 5 × 10^5^ (low-dose) or 20 × 10^6^ (high-dose) *C. neoformans* H99 via the tail vein, the mice were anesthetized, and vascular perfusion was performed. The brain was removed, immediately frozen in optimal cutting temperature (OCT) compound, and cut on a cryostat microtome at a thickness of 8 µm into coated glass slides. The tissue sections were fixed in 4% polyformaldehyde for 10 minutes. The sections were then incubated with 3% goat serum in PBS, followed by incubation with the following antibodies: anti-collagen IV (ab19808, Abcam), anti-cryptococcal polysaccharide (MABF2069, Sigma‒Aldrich), or anti-CD146 (81701S, CST) at 4°C overnight in a humidified chamber. After three washes, the sections were incubated for 1 h with goat anti-mouse IgG (A32723, Invitrogen) to stain the yeast cells in the brain microvasculature and with goat anti-rabbit IgG (A21428, Invitrogen) to delineate the brain microvasculature.

For cultured endothelial cells, the supernatant was removed, and the monolayer cells were washed three times with 1× PBS. The cells were subsequently fixed with 4% formaldehyde for 15 minutes at RT. After being permeabilized with 0.2% Triton X-100 (Yeasen, China) for 15 minutes at RT, the cells were incubated with 1% bovine serum albumin (BSA) for 30 minutes. The cells were subsequently incubated overnight at 4°C with a monoclonal CD146 antibody (81701S, CST) or a polyclonal CD31 antibody (Servicebio, China). After three washes, the cells were incubated for 1 h at RT in the dark with goat anti-rabbit IgG to stain for CD146 in bEnd.3 cells or identify the primary BMECs extracted from the mice.

### Flow cytometry

After stimulation, the adherent bEnd.3 cells were washed twice with PBS to remove free yeast and then digested into a single-cell suspension with 5% pancreatic enzyme. After being washed twice with 1% BSA, the cells were fixed in 4% formaldehyde for 15 minutes at room temperature and permeabilized with ice-cold methanol for 10 minutes. The cells were subsequently incubated with an anti-CD146 antibody (81701S, CST) for 1 hour at RT in accordance with the manufacturer’s instructions. After being washed with 1× PBS three times, the cells were incubated for 30 minutes with AF488-FITC goat anti-rabbit IgG (A32723, Invitrogen) at room temperature in the dark. Flow cytometry was performed via a FACSAria II flow cytometer (BD Biosciences, San Jose, CA, USA); the data obtained were subsequently analyzed via FlowJo (Tree Star, Ashland, OR, USA).

### Cell transfection

bEnd.3 cells were seeded and incubated overnight before transfection. After mixing with Liposomal Transfection Reagent (Yeasen, China) in DMEM without FBS, penicillin, or streptomycin for 25 minutes, the expression plasmid encoding CD146 (MG50794, Sinobiological) was then transfected into bEnd.3 cells at 90%–95% density in DMEM for 48 h. The cells were harvested for further analysis. The blank vehicle plasmid was used as a control.

### Adhesion of *C. neoformans in vitro*

Twenty-four-well plates with 2.5 × 10^5^ endothelial cells per well were seeded, and the culture mixture was maintained at 37°C with 5% CO_2_ for 24–48 h to obtain a fully mature cell monolayer. The cultures were then inoculated with 2 × 10^6^ yeast cells per well, resulting in an approximate infection ratio of 1:3 (target: effector) (40). After infection for 4 h, the nonadherent yeast cells were removed by washing three times with sterile PBS. Then, the endothelial cells were lysed with 400 µL of sterile water for 15 minutes at 37°C to release strongly adhesive cryptococci, and the lysate was plated on Sabouraud’s dextrose agar for CFU counts after 24–48 h of growth at 32°C.

For microscopic counting, after the removal of nonadherent tdTomato-expressing cryptococci via extensive washing, fluorescence microscopy was used to identify adherent yeast cells attached to CD146-overexpressing endothelial cells.

### Tail vein infection and brain CFU

The mice were infected with 5 × 10^5^ (low-dose) or 20 × 10^6^ (high-dose) *C. neoformans* H99 via the tail vein. Twenty-four hours postinfection, the mice were sacrificed and perfused. The brains were dissected and homogenized in sterile water. Appropriate dilutions of the homogenates were plated onto Sabouraud’s dextrose agar plates, and CFUs were enumerated after 24–48 h of growth at 32°C.

### Neutralizing antibody treatment

The mice were intraperitoneally (*i.p*.) treated with 100 µg of AA98 (a gift from Dr. Xiyun Yan and Dr. Hongxia Duan) or mouse IgG (36111ES, Yeasen) per pop before being infected with 10 × 10^6^ *C. neoformans* via the tail vein. After 24 h of infection, the mice were sacrificed and perfused. After dissection, the brains, lungs, and spleen were homogenized in sterile water. Appropriate dilutions of the homogenates were plated onto Sabouraud’s dextrose agar plates, and the CFUs were enumerated after 24–48 hours of growth at 32°C.

For survival experiments, the mice were i.p. treated with 100 µg of AA98 or the isotype IgG before being infected with 10 × 10^6^ *C. neoformans* via the tail vein. Mice were observed over the course of infection. Any mouse in a moribund state and/or distress was euthanized to avoid unnecessary suffering.

### Statistical analysis

Data were subjected to testing for a normal distribution. All the data are expressed as the means ± SEM, and all the statistical analyses were performed via GraphPad Prism 8. Single comparisons were conducted via unpaired *t* tests. Multiple comparisons were tested by using one-way ANOVA with Tukey’s adjustment. And the survival study comparison was performed using the Log-rank (Mantel-Cox) test. For all analyses, statistical significance was set as *P < 0.05; **P < 0.01; ***P < 0.001; ****P < 0.0001; and ns, not significant.

## Data Availability

The data sets used and/or analyzed during the current study are available from the corresponding author upon reasonable request.

## SUPPLEMENTAL MATERIAL

Fig. S1 iai.00145-25-s0001.docx A baseline (no infection) control CD146 immunofluorescence image of brain tissue.

ASM does not own the copyrights to Supplemental Material that may be linked to, or accessed through, an article. The authors have granted ASM a non-exclusive, world-wide license to publish the Supplemental Material files. Please contact the corresponding author directly for reuse.
