# Supplementary material for: Involvement of CD146 in the Cryptococcus neoformans adhesion and infection of brain endothelial cells
Source: Infect Immun. 2025 Apr 10;93(5):e00145-25. doi: 10.1128/iai.00145-25 (PMC12070736; doi:10.1128/iai.00145-25)
Supplement: Fig. S1 — A baseline (no infection) control CD146 immunofluorescence image of brain tissue. [file iai.00145-25-s0001.docx]

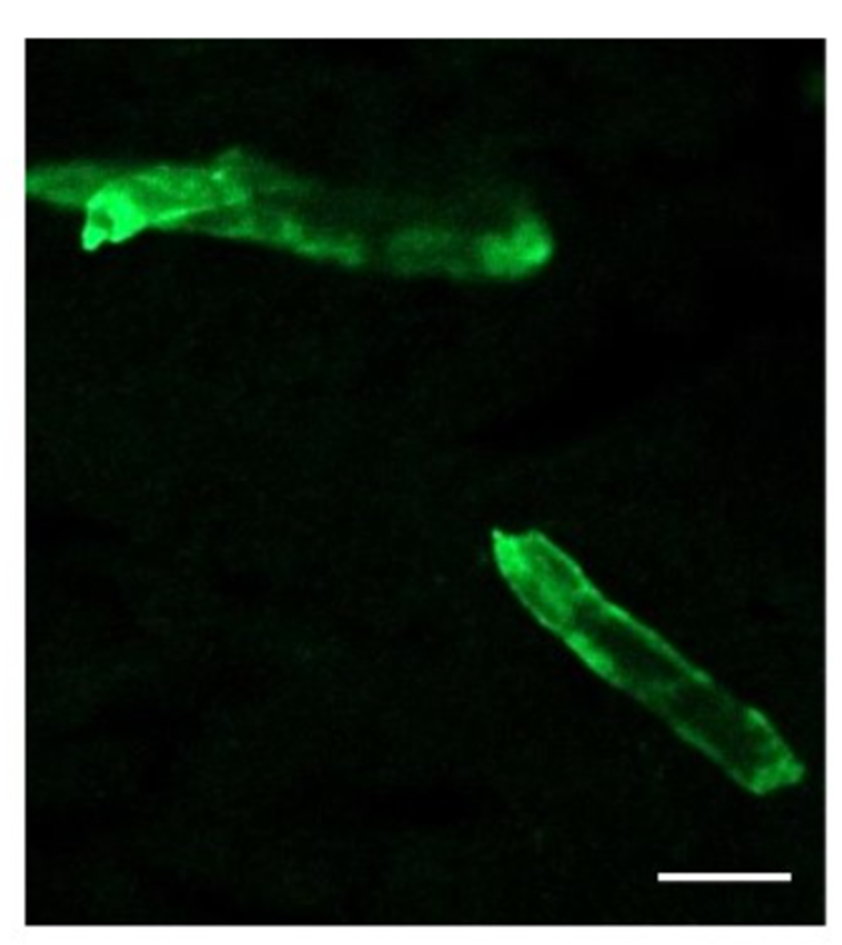


Supplementary Fig. 1 A baseline (no infection) control CD146 immunofluorescence image of brain tissue was performed. Scale bar: 10 μm.
